# Impact of Load-Related Neural Processes on Feature Binding in Visuospatial Working Memory

**DOI:** 10.1371/journal.pone.0023960

**Published:** 2011-08-24

**Authors:** Nicole A. Kochan, Michael Valenzuela, Melissa J. Slavin, Stacey McCraw, Perminder S. Sachdev, Michael Breakspear

**Affiliations:** 1 Brain and Ageing Research Program, School of Psychiatry, Faculty of Medicine, University of New South Wales, Sydney, Australia; 2 School of Psychiatry, Faculty of Medicine, University of New South Wales, Sydney, Australia; 3 Neuropsychiatric Institute, Prince of Wales Hospital, Sydney, Australia; 4 Regenerative Neuroscience Group, School of Psychiatry, Faculty of Medicine, University of New South Wales, Sydney, Australia; 5 Dementia Collaborative Research Centre, School of Psychiatry, Faculty of Medicine, University of New South Wales, Sydney, Australia; 6 The Black Dog Institute, Sydney, Australia; 7 Queensland Institute of Medical Research, Brisbane, Australia; 8 Royal Brisbane and Women's Hospital, Herston, Australia; University of Regensburg, Germany

## Abstract

**Background:**

The capacity of visual working memory (WM) is substantially limited and only a fraction of what we see is maintained as a temporary trace. The process of binding visual features has been proposed as an adaptive means of minimising information demands on WM. However the neural mechanisms underlying this process, and its modulation by task and load effects, are not well understood.

**Objective:**

To investigate the neural correlates of feature binding and its modulation by WM load during the sequential phases of encoding, maintenance and retrieval.

**Methods and Findings:**

18 young healthy participants performed a visuospatial WM task with independent factors of load and feature conjunction (object identity and position) in an event-related functional MRI study. During stimulus encoding, load-invariant conjunction-related activity was observed in left prefrontal cortex and left hippocampus. During maintenance, greater activity for task demands of feature conjunction versus single features, and for increased load was observed in left-sided regions of the superior occipital cortex, precuneus and superior frontal cortex. Where these effects were expressed in overlapping cortical regions, their combined effect was additive. During retrieval, however, an interaction of load and feature conjunction was observed. This modulation of feature conjunction activity under increased load was expressed through greater deactivation in medial structures identified as part of the default mode network.

**Conclusions and Significance:**

The relationship between memory load and feature binding qualitatively differed through each phase of the WM task. Of particular interest was the interaction of these factors observed within regions of the default mode network during retrieval which we interpret as suggesting that at low loads, binding processes may be ‘automatic’ but at higher loads it becomes a resource-intensive process leading to disengagement of activity in this network. These findings provide new insights into how feature binding operates within the capacity-limited WM system.

## Introduction

Capacity constraint is a fundamental characteristic of working memory (WM). For visual WM, capacity limits have been estimated to be between three and five items [1], [2]. How the brain accommodates this restricted capacity is not well understood. One proposal is that integration or ‘binding’ of separate aspects of the environment is employed as an adaptive method of information compression [2], [3]. Yet exactly how this is achieved remains a central question about brain function even after decades of research [4]. In recent years, functional magnetic resonance imaging (fMRI) has been used to investigate the neural correlates that underlie our ability to bind separate aspects of visual information and maintain this unified form in WM. In this study, we investigate how increasing WM load (i.e. the number of items to be remembered) influences binding-related cortical processes.

Several types of binding have been described [for reviews see 4,5]. At the most basic visual perceptual level, two or more features are bound together to form a unified object representation, a process thought to occur automatically when objects are attended to [6]. Electrophysiological studies suggest this may be mediated by temporally synchronous high frequency oscillations of feature selective cells within early visual cortex [7]. Integrated representations can also be maintained for brief periods in visual WM for explicit recall and recognition [8]. Cognitive researchers have suggested that binding in WM may require controlled attention or executive processes which may be resource-demanding [9], [10]. The most recent revision to Baddeley's influential *multicomponent model* of WM has included an episodic buffer responsible for the formation, temporary storage and retrieval of bound representations [11]. It has been hypothesised that active cognitive processes controlled by the Central Executive may be needed to perform these functions [9]. Studies investigating the neural correlates of binding in WM have observed greater activity in regions in the prefrontal cortex (PFC) [3], [12], [13], hippocampus [12], [14], [15], superior parietal cortex and intraparietal sulcus [16, but see 17] when a task demands binding of features (i.e. feature conjunctions) compared to when features are encoded or maintained separately. These findings support the proposition that specific higher cognitive processes may be needed to create and maintain feature binding in WM [9]. A recent study, however did not support this contention since cortical responses were reduced during feature binding in lateral dorsal and ventral PFC, regions typically activated when component features are presented alone [18]. The findings were interpreted within a *modified biased competition model* [19] whereby in the feature binding condition, biasing signals flow between dorsal (spatial) and ventral (object) regions of the PFC, biasing competition within each region and resulting in an overall decrease in regional activity.

Research findings are therefore not conclusive as to whether binding of features in WM engages specific cognitive processes not otherwise involved in memory for simple features. Thus the question remains whether binding in WM is a resource-demanding process. If it is resource-demanding, it follows that binding may be influenced by factors that also place demands on the limited capacity WM system such as memory load. Interestingly, although brain responses associated with manipulations of memory load are typically distributed and partly dependent on task specific features (e.g. verbal and spatial), PFC [20] and parietal cortex [21], [22] appear to be key regions and hippocampus also has a load-dependent function [23], [24]. Hence a common set of brain regions may be integral to feature binding and load in WM, potentially pointing to a common mechanism underlying both. This has not been empirically tested to date. Therefore, it is not known how these fundamental cognitive processes and their distributed cortical responses interact.

The aim of this study was to examine the effect of WM load on the neural correlates of feature binding. Using a customised, factorially-designed visuospatial WM task in which WM load and binding (via feature conjunction versus single feature effects) were manipulated independently, we sought to examine the separate effects of binding and load and their potential interaction which in turn may improve our understanding of how binding relates to WM capacity limitations. Existing cognitive and neurobiological models of feature binding make different predictions about the relationship between binding and load. According to Baddeley's cognitive model, binding may be resource-demanding if active processes are engaged [9] so that when load is increased, binding and load would be expected to jointly place greater demands on the limited capacity system. This model predicts an interaction of task demands for binding and load that would be expressed as sub-additive responses in PFC, parietal cortex or hippocampus, and lower task accuracy for feature conjunctions compared to single features. In contrast, the neural synchrony and biased competition models suggest that feature binding is not resource-demanding since it simply engages regions that subserve the component features. According to these neurobiological models, load should equally modulate feature conjunction and single feature responses therefore no interactions are predicted for neural or behavioural responses. Rather, where effects of binding and load are represented in overlapping cortical regions, their conjunction should be purely additive. Using an event-related fMRI paradigm, we aimed to characterise the relationship of binding and load during each of the sequential phases of encoding, maintenance and retrieval. Interestingly, we observed that this relationship qualitatively differed through each phase of the WM task and its cortical expression was represented in a diverse set of brain areas.

## Materials and Methods

### Ethics statement

Participants gave written informed consent and the study was approved by the University of New South Wales Human Research Ethics Committee.

### Participants

Nineteen young, healthy, right handed participants (mean age 26.8, SD 4.3 years; mean education 15.8, SD 2.0 years; 9 female) were recruited via advertisement. Data from one participant was excluded due to misinterpretation of instructions. Participants denied any history of neuropsychiatric disorders or recent illicit substance use and were not taking psychoactive medications.

### Paradigm

**Figure 1:**
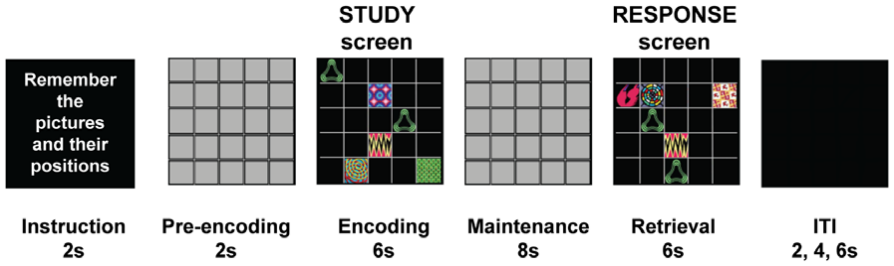
Paradigm sequence, stimuli and timing in a single trial. Schematic representation of an example of a single ‘Medium’ load true positive trial in the Combined task. Each box represents a trial component with duration of each denoted (in seconds). Each fMRI trial consisted of a) an instruction screen indicating task component to be remembered (either Picture, or Position, or Picture *and* Position); b) a pre-encoding fixation grid; c) a study screen during which sets of targets were presented for participants to remember (encoding phase); d) a fixation mask consisting of a grid masked with grey squares to minimise perceptual after-effects (maintenance phase); e) a response screen during which another set of stimuli were displayed and participants indicated with a button press (yes/no) if any *one* of the targets had been present in the immediately preceding study screen (retrieval phase) and f) the inter-trial interval with duration jittered. Multicoloured abstract designs represent target stimuli to be remembered (either the picture itself; or the position in which the picture is placed; or both). Curved green shape represents non-target (filler) items.

Participants performed a delayed recognition visuospatial WM paradigm featuring three tasks; Picture, Position and Combined *(feature conjunction of picture in position).* Figure 1 depicts the events and timing of a single fMRI trial. Participants viewed a *study* screen consisting of a 5×5 grid on which target pictures and non-target filler items were presented. Picture stimuli consisted of 154 abstract, multi-coloured designs obtained from an online database (Barbeau, E.J.: http://cerco.ups-tlse.fr/~barbeau/). While verbalisation of the stimuli was possible, the use of abstract designs rather than everyday objects lessened the likelihood that subjects could use an easy naming strategy to distinguish items at encoding and retrieval. Participants were asked to remember the target pictures only (not the positions) in the Picture task, the positions that target pictures appeared in (not the pictures) in the Position task and both, that is, target pictures and the positions they appeared in, for the Combined task. A delay period of eight seconds followed during which a fixation mask (5×5 grid with no stimuli) was presented. Finally, a *response* screen, consisting of the grid and another set of stimuli (targets and fillers), was presented. Participants were asked to respond via button press (yes/no) to the question of whether any *one* of the target stimuli on the response screen had been present in the immediately preceding study screen. Four types of response probe trial were presented at retrieval for all tasks: (1) *No match*: no picture or position is repeated, (2) *Picture only match:* one target picture is repeated in a different position, (3) *Position only match:* one target position is repeated with a different picture and (4) *Both match:* one target picture is repeated in the same position it was originally presented in (i.e. same pairing of picture and position). For ‘match’ trials, stimuli other than the target item were new. For the Combined task only trials of type (4) constituted a match trial, the three other probe types were non-match trials thereby precluding a single-feature-based strategy in the Combined feature task.

#### Setting of load levels

WM load was manipulated by altering the number of target stimuli relative to non-target (filler) stimuli presented for encoding. Filler items (non-descript curved green shape) were incorporated into Study and Response screens to hold overall visual input constant at six items (targets plus fillers) for all load conditions. Appropriate load levels for the High load were determined prior to the scan on a subject-wise basis to reduce expected inter-subject variance and to avoid floor effects by ensuring all subjects were performing above chance. Target accuracy for the High load was approximately 60–70% translating to either 5 or 6 target stimuli (a maximum of 6 targets was possible). Most participants received 6 targets with two receiving 5 targets. Medium load was universally set at four targets after pilot testing showed that this provided a challenge with an accuracy of 75–85% correct and minimal inter-subject variance. Low load was universally set at one target.

### Study Design

A 3 (task) ×2 (load) repeated measures factorial design was used to investigate the separate main effects of task component and WM load, and their interaction. The physical properties of the stimulus array (pictures and fillers placed in a spatial grid) presented at encoding and retrieval were identical for all tasks, thereby allowing comparison of task-specific cognitive processes while controlling visual input. Since the purpose of the experiment was to examine comparative feature conjunction and load effects, no explicit baseline task was employed. The principal study analyses were restricted to two levels of load common to all tasks (Low, Medium). Additionally, we were interested in examining the neural correlates of load increases beyond proposed visual WM capacity limits [1]. Due to time constraints, the additional supracapacity load (High load) was only present for the Combined task.

The three tasks were conducted in separate scan sessions with a short break (3–5 minutes) between each to remind participants of the next task's instructions. Participants performed a total of 98 trials (14 trials for each task at each level of difficulty; nine were match trials). Intertrial intervals were jittered pseudorandomly between two and six seconds to temporally decorrelate the evoked haemodynamic responses between trials. Task order, load order (ascending or descending load) and button press (left/right for yes) were independently counterbalanced across subjects. A practice session was provided prior to the scan wherein practice trials were administered until participants reached a criterion of five or more correct from a maximum of six trials. During the scanning session, the visual stimuli were displayed on a rear projection screen and viewed by participants through a mirror attached to the head coil. Foam padding was used to minimize head motion and ear plugs and headphones were used to reduce scanner noise.

### Imaging protocol

Functional T_2_*-weighted echoplanar images (EPI) were acquired on a Philips (Intera) 3.0 Tesla scanner with an 8-channel SENSE head coil using an interleaved sliced acquisition sequence (29 axial slices, repetition time (TR)  = 2000 ms, echo time (TE)  = 30 ms, 90^0^ flip angle, matrix size = 112x128, field of view (FOV)  = 240 mm, voxel size  = 2.14×2.73, slice thickness  = 4.5 mm, 0 mm slice gap). One run of each task was acquired consisting of 383, 381, 568 whole brain volumes for the Picture, Position and Combined tasks respectively.

### Behavioural data analysis

Given the asymmetry of true positive to true negative trials (approximately 65%:35%), statistical analyses of accuracy data were performed using dPrime statistic (d') to control for potential affirmative response bias. d' is estimated from the hit rate (true positives) and the false alarms and provides a measure of sensitivity to the ‘signal’ with a higher value representing better discrimination. d' and response time (RT) were subjected to a 3 (task) ×2 (load) repeated measures analysis of variance (ANOVA), and a separate repeated measures ANOVA examined three levels of load in the Combined task. Significant main effects and interactions were further investigated post-hoc, using paired samples t*-*tests and applying a Bonferroni correction of *p*<0.05. Trials with no response recorded within 6000ms (0.1% of trials) were not included in statistical analyses.

### Imaging data analysis

fMRI images were processed and statistically analysed using SPM5 software (The Wellcome Trust Centre for Neuroimaging at University College London, UK: http://www.fil.ion.ucl.ac.uk). Preprocessing included realignment of the time series to the first image using a 6-parameter rigid-body transformation; spatial normalization via registration of the mean EPI image into standard [Montreal Neurological Institute (MNI)] space (MNI/CBM avg152 T2* template) using a 12-parameter affine transformation, resampling into 3×3×3 mm isotropic voxels, and spatial smoothing of the normalised images using an 8mm full-width-half-maximum (FWHM) Gaussian kernel.

The experiment used a mixed event-related/blocked functional MRI design allowing relative temporal disambiguation of the neural correlates associated with task demand, load level, and memory phase (encoding, maintenance and retrieval). Statistical analysis of the time series of images was conducted using the General Linear Model (GLM) [25] with regressors modelling each of the task, load and memory phase components as a 100ms delta function at the onset of each, convolved with the canonical haemodynamic response function. The model estimated four trial components - pre-encoding, encoding, maintenance and retrieval, and the realignment parameters to control for movement-related variability. A high-pass filter of 128 seconds was used to remove low frequency noise. For each participant, t-contrasts on BOLD signal changes were defined for individual events of interest combining a single task and load level (e.g. Picture task/ Low load). Images were examined for movement and susceptibility artifacts.

Group-level, random-effects analyses were performed by entering individual subject BOLD contrast images into a 3 (task) ×2 (load: Low vs Medium) flexible factorial ANOVA including a subject factor and non-sphericity correction for repeated measures. Separate ANOVAs were conducted for each of the three trial phases. All trials were included in the general linear model. A supplementary analysis was performed using correct trials only. F-statistics testing for main effects of task and load, and the interaction were thresholded voxel-wise using family-wise error (FWE) correction to control for multiple comparisons across the whole brain [26]. Planned t-tests were subsequently performed. T-maps were initially thresholded at *p*<0.001 (uncorrected) and only clusters significant at *p*<0.05 (FWE-corrected for multiple comparisons) are reported. A ROI analysis in the hippocampus was performed within a bilateral hippocampal mask (561 voxels) defined using the Wake Forest University Pick Atlas toolbox for SPM [27]. Inferences about significant activity were based on local maxima statistics surviving FWE correction. This method was used to maximise power to detect potentially smaller effects in this brain region due to susceptibility-induced signal reduction [28].

The main focus of the study was the investigation of task x load interactions that reflected modulation of feature conjunction activity by load. The following interaction contrasts were formulated: i) *(Combined > Picture + Position) x (Medium > Low)* and ii) *(Combined < Picture + Position) x (Medium > Low)* to examine for changes in Combined task activity (increased and decreased) relative to averaged single feature activity, under conditions of increased load. Of secondary interest, we investigated regions where activity in the Combined task was increased or decreased relative to both single feature tasks, *independent* of load using the following contrasts: i) *Combined > (Picture + Position)* and ii) *Combined < (Picture + Position)*. This analysis was performed to quantify the nature of any non-interactive effects (such as linear addition of load and binding in shared cortical areas) and to ensure that task effects in our data accord with what is generally described in the literature. Contrasts incorporating ‘Combined > or < Position + Picture’ were mathematically operationalized by contrasting parameter estimates for the Combined task with the average of the single feature tasks, namely (Picture + Position)/2. This formulation conforms to the intuitive notion that an area showing a specific binding effect will show activity that is greater (or weaker) than both of the single feature tasks and significantly greater (or weaker) than their average. As a supplementary analysis, single task subtractions (e.g. *Combined > Position, Combined > Picture)* were performed to allow comparison with previous studies [13], [18] although these contrasts alone cannot completely disentangle potential binding effects from feature effects related to the non-subtracted component.

The three levels of load (Low, Medium, High) for the Combined task were modelled in a separate flexible factorial ANOVA and the same statistical thresholds were used for the F- and t- statistics as detailed above.

## Results

### Behavioural performance

**Figure 2:**
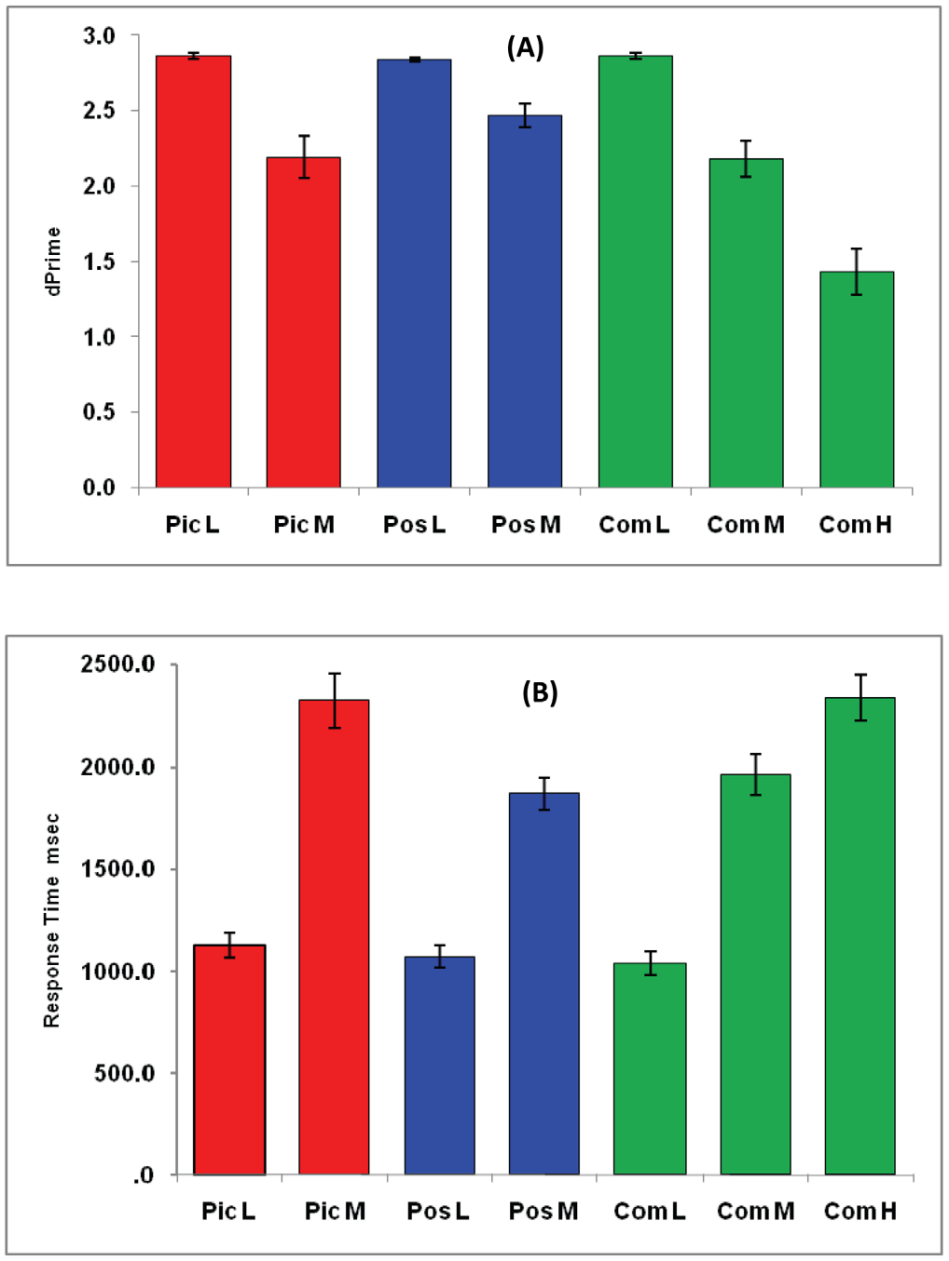
Behavioural data. L =  Low load, M =  Medium load, H  =  High load, Pic  =  Picture task, Pos  =  Position task, Com  =  Combined task. Top figure shows mean d-Prime and lower figure shows mean response time (in milliseconds) for each task and load. Error bars represent ±1 standard error of the mean.

Mean dPrime (accuracy) and RT for each task and load are depicted in Figure 2. Strong main effects of load were found for accuracy [*F*(1,17)  = 59.05, *p*<0.001] and RT [*F*(1,17)  = 287.0, p<0.001). For accuracy, no significant difference for task [*F*(2,34)  = 1.61, *p* = 0.21] was observed. However there was a non-significant trend for a task x load interaction [*F*(2,34)  = 2.56, *p* = 0.09]. As seen in Figure 2, accuracy declined in all tasks with increased load. For RT, a significant effect of task [*F*(1.52, 25.78)  = 11.35, *p* = 0.001; Greenhouse-Geisser correction for non-sphericity] and a significant task by load interaction [*F*(1.29,21.96)  = 6.97, *p* = 0.01; Greenhouse-Geisser correction for non-sphericity] were found. Figure 2 depicts greater increase in RT from Low to Medium for the Picture task compared to the other two tasks (*p*<0.05). Further examination however, revealed the effect was present in true negative but not true positive trials suggesting that slower response times may have resulted from the need to engage in more detailed processing of the complex pictures, compared to using simpler spatial configurations to reject true negatives in the Position task and in some trials of the Combined task. There were no significant effects of task order or load order (ascending or descending) on performance measures.

Separate analyses of the Combined task revealed strong load effects over the three load levels for accuracy [*F*(2,34)  = 46.77, *p*<0.001] and RT [*F*(2,34)  = 125.01, *p*<0.001] and significant differences between each load for both measures (*p*<0.01).

### Functional imaging

Following the factorial design of the experiment, load effects are presented followed by task effects, with a focus on comparisons between the Combined task and single feature tasks to reveal potential binding effects, and finally the analysis of their interaction. Within each analysis we consider the three phases of each WM trial namely encoding, maintenance and retrieval. The experiment was designed principally to examine contrasts and interactions across task conditions and loads. We therefore did not undertake direct contrasts between phases of the same trials. Rather, contrasts were conducted within phases (encoding, maintenance, retrieval) across the conditions of task and load. However, in order to allow an informal and cautious comparison of these different contrasts, time course plots for group-averaged event-related activity were examined for each task over a trial for a number of brain regions. Inspection of exemplar BOLD responses from supra-threshold voxels and comparison with time series obtained by formally integrating predicted neurovascular dynamics across simulated trials with known effects, suggest satisfactory differentiation between trial phases where they truly differ (Figure S1). This suggests that cautious inferences can be made about differences in phase-specific activity between conditions (see Discussion for further consideration of this issue).

The factorial ANOVA analyses revealed significant main effects for task and load at each trial phase, and a single interaction effect limited to the retrieval phase. Table S1 provides a full listing of significant results for main effects and the interaction. The following sections report findings from hypothesis-driven t-contrasts to explore the direction of the effects underlying each significant F-contrast.

**Load effects.** Load-related activity was investigated by comparing Medium and Low loads, in both directions (Medium < > Low). Load effects were strong and differentially distributed over two large sets of brain regions that either increased or decreased their brain activity in response to increasing load. These general patterns were observed for all task phases with some variability in the specific distribution of regions engaged during the WM trial. Activity *increased* as a function of increasing load in occipitotemporal, parietal, frontal, subcortical and cerebellar regions, and right hippocampus (upon ROI analysis) consistent with previous observations of load-related activity in visual WM tasks [23], [29]–[31] (Table 1: *Medium>Low*). Activity *decreased* as a function of increasing load in lateral regions of parietal, temporal and frontal cortex, insula and also medial regions of frontal cortex and posterior cingulate, the latter corresponding to a network of brain regions described as part of the default mode network (DMN) [32] (Table 1: *Medium < Low*). Group-averaged load-related activity maps and plots of the parameter estimates (*β* coefficients) depicting average task and load effect sizes in selected brain regions are shown in Figure S2.

**Table 1:** Regions of significant activity related to working memory load.

		MNI coordinates						
Analysis	Brain region	Right/left	*x*	*y*	*z*	*T*-value	Cluster size	Brodmann Area
**Medium > Low**								
*Encoding*	Lingual Gyrus	R	27	−75	−12	10.76	3133	19
	Superior Frontal	L	−24	−3	57	9.98	1466	6
	Inferior Frontal	R	45	3	27	4.53	68	9
	Cerebellum	R	6	−30	−9	7.32	168	
	Caudate	L	−15	12	6	7.29	365	
	Putamen	R	18	15	−9	7.08	316	
	Hippocampus	*R	21	−30	−9	4.08	11	
*Maintenance*	Precuneus	L	−18	−57	57	9.84	2475	7
	Middle Frontal	L	−24	−6	54	9.76	1854	6
		L	−30	42	12	5.08	96	10
		R	39	39	27	5.39	113	10
	Cerebellum	L	−33	−60	−36	5.22	202	
	Caudate	L	−15	6	9	5.85	357	
	Putamen	R	24	18	3	5.79	295	
*Retrieval*	Cuneus	L	−27	−78	27	9.29	1025	19
	Lingual Gyrus	L	−27	−81	−15	5.85	117	18
	Fusiform	R	27	−63	−12	4.88	121	19
	Cerebellum	R	6	−30	−9	5.49	71	
	Middle Frontal	L	−24	0	57	7.99	961	6
		R	30	−3	57	6.52	249	6
	Inferior Frontal	R	33	24	−6	6.74	94	47
		R	42	6	30	4.95	74	9
		L	−30	24	−3	5.63	55	47
	Caudate	L	−9	6	0	4.42	92	
**Medium < Low**								
*Encoding*	Supramarginal Gyrus	R	60	−54	36	7.15	354	40
	Middle Temporal	R	60	−36	−6	4.99	89	21
	Middle Frontal	L	−42	15	42	5.35	52	9
	Medial Frontal	L	−3	39	39	4.88	94	6
	Insula	R	45	6	0	5.80	641	13
*Maintenance*	Angular Gyrus	R	57	−60	33	4.67	99	40
	Medial Frontal	L	−6	54	24	5.07	596	9
	Precentral Gyrus	R	48	−15	12	4.89	336	13
	Insula	L	−42	−15	18	5.42	275	13
	Posterior Cingulate	R	6	−51	24	4.10	92	23
	Cingulate	L	−6	−12	45	4.05	53	31
*Retrieval*	Cuneus	R	3	−81	33	5.10	140	19
	Superior Temporal	L	−54	−30	15	4.29	81	42
	Superior Frontal	R	18	54	36	4.95	79	9
	Inferior Frontal	R	51	42	6	4.61	65	46
	Cingulate		0	−21	39	6.66	538	24
	Insula	R	42	6	6	6.46	1385	
		L	−39	−15	21	5.18	338	

List of significant clusters for load comparisons (averaged over task) for encoding, maintenance and retrieval. Standardized Montreal Neurological Institute (MNI) co-ordinates represent peak maxima of significant clusters (family-wise error (FWE)-corrected threshold). Approximate Brodmann areas are listed.

*Hippocampus cluster was significant upon ROI analysis. Threshold was applied voxel-wise (FWE-corrected) to the region identified with a bilateral hippocampal mask defined using Wake Forest University Pick Atlas.

Supracapacity load effects in the Combined task were examined via High load comparisons, however no significant activity was observed in comparisons between Medium and High loads (*High > Medium and High < Medium*). Load effects (both positive and negative) were attenuated beyond the Medium load (Figure S2).

**Task effects.** With regard to task effects, our primary focus was to identify Combined task (feature conjunction) activity that may potentially represent binding processes. By subtracting the averaged activity for both single feature conditions (*Combined > Picture + Position)* we sought specific effects related to the Combined task beyond those effects that could be attributed to performance of each of the component single feature tasks. Activity related to single feature effects is listed in Table S2. Briefly, this analysis revealed the expected neural correlates for Position > Picture and Picture > Position comparisons, that is, patterns of activity largely corresponded to the classical ventral/object-dorsal/spatial visual processing segregation [33] particularly for the encoding phase.

**Figure 3:**
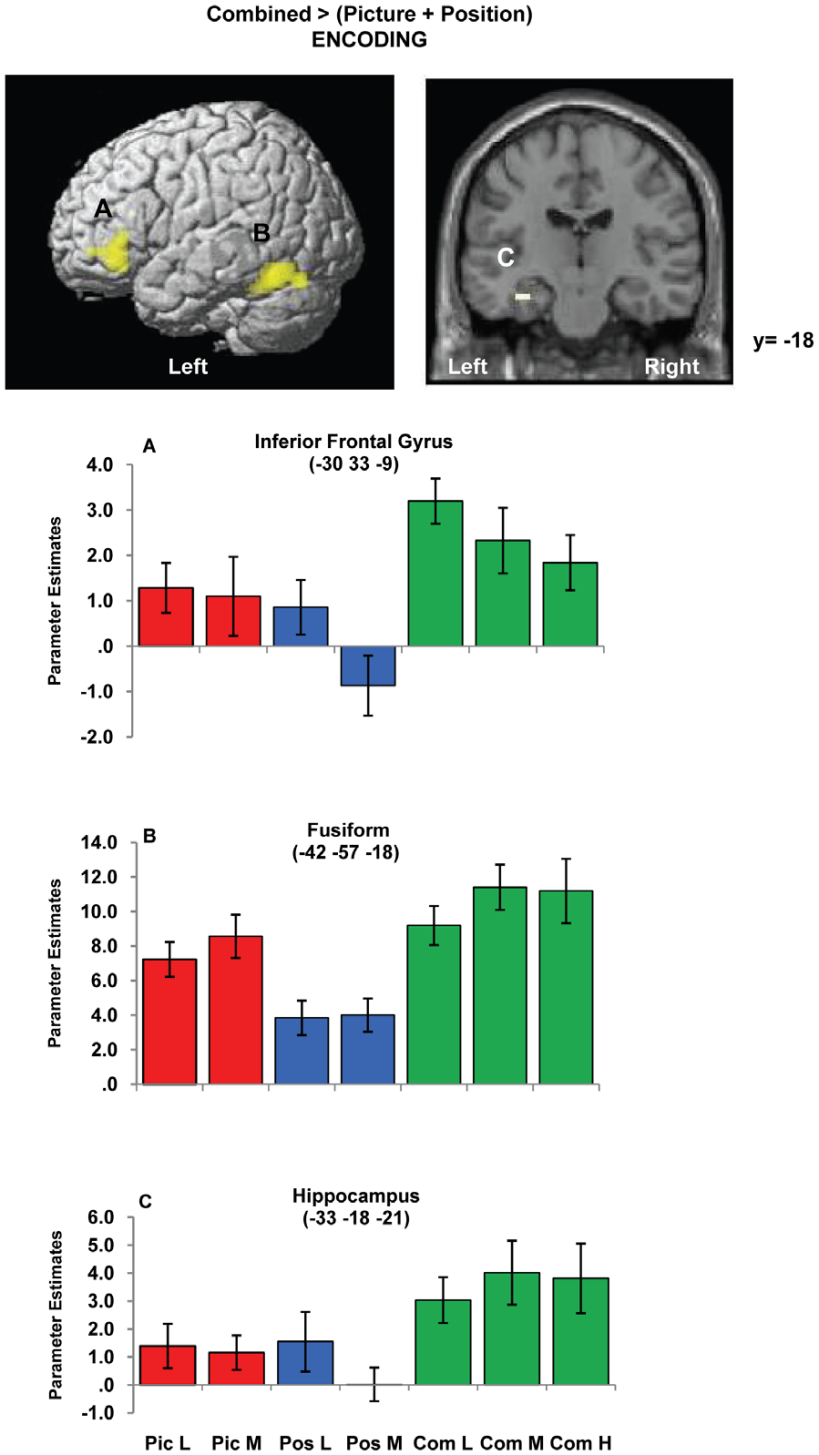
Combined task-related activity during encoding. L =  Low load, M =  Medium load, H  =  High load, Pic  =  Picture task, Pos  =  Position task, Com  =  Combined task. Left top image shows group mean activity for the contrast ‘Combined >(Picture + Position)’ for the encoding phase and is superimposed on the SPM rendered brain image. Activity depicted is significant using a cluster-defining whole brain threshold of p<0.001 and FWE (p<0.05) cluster correction. Right top image shows significant left hippocampal activity using region-of interest analysis (FWE p<0.05 corrected applied voxel-wise to bilateral hippocampal mask). Increased activity was observed for the Combined task relative to the averaged single feature tasks at equal load levels in these regions. Plots of the mean parameter estimates (β coefficients) for each task and load condition are provided for peak voxels of the identified suprathreshold clusters. Beta estimates for the High load are also included to depict relative effects between load levels in the Combined task, however the main task effects were based on Low and Medium loads. Error bars represent ±1 standard error of the mean.

*Combined > (Picture + Position):* At encoding, greater activity was observed in left fusiform, left inferior frontal cortex and left hippocampus (upon ROI analysis) for the Combined task relative to averaged single feature tasks (Table 2 and Figure 3). Parameter estimate plots (Figure 3) show that Combined task activity was relatively stronger than both Picture and Position task activity at Low and Medium loads, in inferior frontal gyrus and hippocampus. In the fusiform region however, Combined task activity was stronger than Position activity but comparable to Picture activity and therefore likely reflected feature extraction demands common to the Combined and Picture tasks but absent in the Position task.

**Table 2:** Regions of significant activity for the combined task relative to both single feature tasks; independent of load, and interacting with load.

		MNI coordinates						
Analysis	Brain region	Right/left	*x*	*y*	*z*	*T*-value	Cluster size	Brodmann Area
**Combined > (Picture + Position)**								
*Encoding*	Fusiform	L	−42	−57	−18	5.12	72	37
	Inferior Frontal	L	−30	33	−9	4.64	51	47
	Hippocampus*	L	−33	−18	−21	3.73	3	
*Maintenance*	Superior Occipital	L	−30	−75	27	5.53	111	19
	Precuneus	L	−21	−57	42	4.22	63	7
	Superior Frontal	L	−27	−3	63	4.41	67	6
**{Combined < (Picture + Position)} x (Medium > Low)**								
*Retrieval*	Cingulate	L	−3	−27	33	6.39	653	23
	Caudate	R	21	15	15	5.46	169	
		L	−18	24	3	5.03	131	
	Cerebellum	L	−9	−45	−33	4.67	86	
	Superior Temporal	R	63	0	−6	4.01	64	21

List of significant clusters for the following comparisons: i) Combined < > average (Picture + Position) averaged across Low and Medium loads and ii) Combined < > average (Picture + Position) x (Medium > Low). No significant activity was observed for Combined > average (Picture + Position) at retrieval or for the reverse contrast: Combined < average (Picture + Position) at any trial phase. A significant task x load interaction was observed at the retrieval phase for {Combined < (Picture + Position)} x (Medium > Low); no significant activity was observed for the reverse interaction contrast: {Combined > (Picture + Position)} x (Medium > Low) at retrieval. Standardized Montreal Neurological Institute (MNI) co-ordinates represent peak maxima of significant clusters (family-wise error (FWE)-corrected threshold). Approximate Brodmann areas are listed.

*Hippocampus cluster was significant upon ROI analysis. Threshold was applied voxel-wise (FWE-corrected) to the region identified with a bilateral hippocampal mask defined using Wake Forest University Pick Atlas.

**Figure 4:**
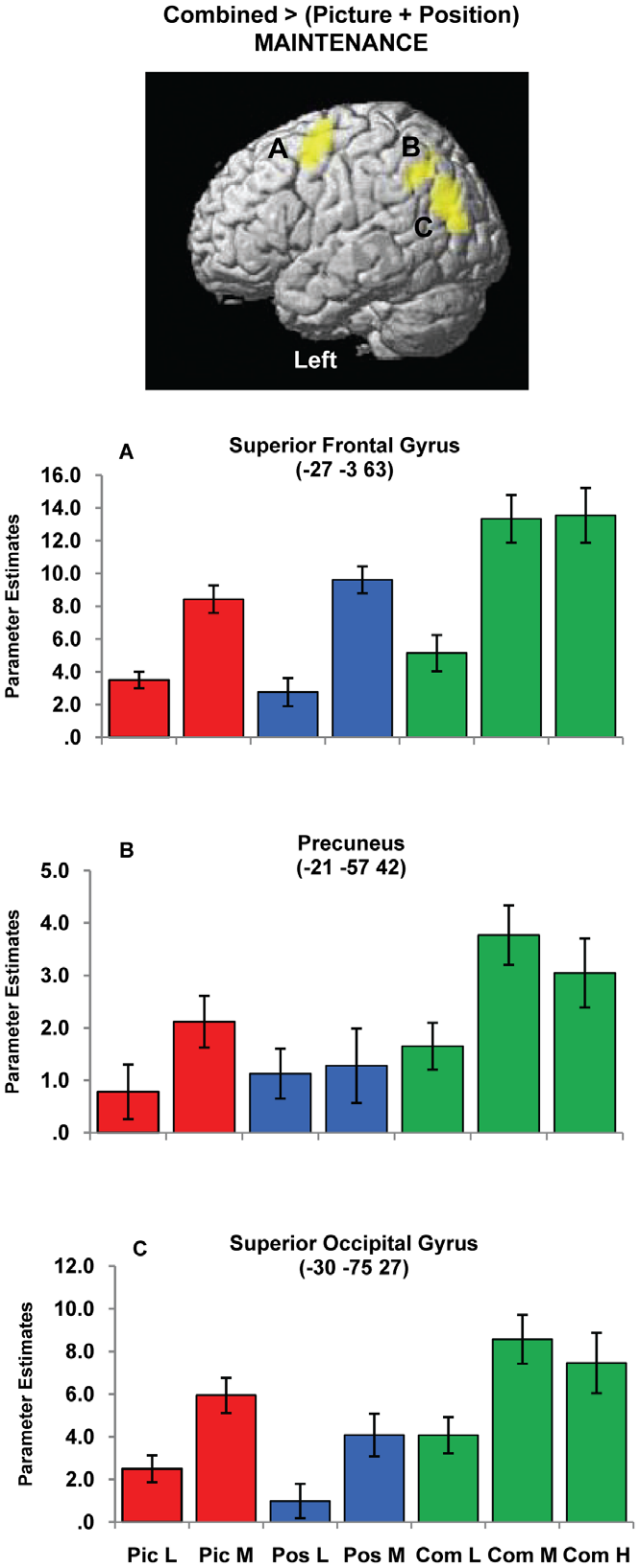
Combined task-related activity during maintenance. L =  Low load, M =  Medium load, H  =  High load, Pic  =  Picture task, Pos  =  Position task, Com  =  Combined task. Image shows group mean activity for the contrast ‘Combined >(Picture + Position)’ for the maintenance phase and is superimposed on the SPM rendered brain image. Activity depicted is significant using a cluster-defining whole brain threshold of p<0.001 and FWE (p<0.05) cluster correction. Increased activity was observed for the Combined task relative to the averaged single feature tasks at equal load levels in these regions. Plots of the mean parameter estimates (β coefficients) for each task and load condition are provided for peak voxels of the identified suprathreshold clusters. The beta estimates for the High load are also included to depict relative effects between load levels in the Combined task, however the main task effects were based on Low and Medium loads. Error bars represent ±1 standard error of the mean.

**Figure 5:**
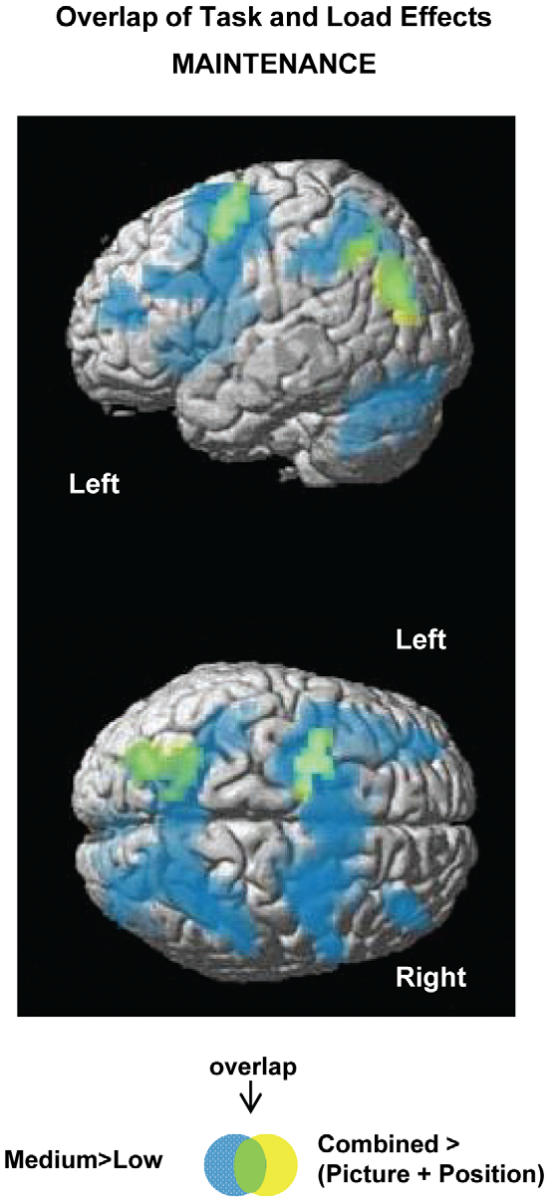
Overlap of task and load effects during maintenance. Image shows group mean activity for the contrasts: [Combined >(Picture + Position):yellow] and load-related activity [Medium>Low: blue] for the maintenance phase superimposed on the SPM rendered brain image. Activity depicted is significant using a cluster-defining whole brain threshold of p<0.001 and FWE (p<0.05) cluster correction. Overlap of common brain regions is depicted in green.

For the maintenance phase, greater activity was observed for the Combined task relative to averaged single feature tasks in left lateralised regions of superior occipital, precuneus and superior frontal cortex (Table 2 and Figure 4). Inspection of task effects (Figure 4) suggests stronger Combined task activity relative to each of the single feature tasks in all three regions and also an apparent load effect *(Medium > Low)* for each of these regions. The possibility of an overlapping, additive effect of Combined versus single features, and load, was investigated by performing a statistical conjunction test between the separate contrasts using the approach advanced by Nicholls et al. [34]. The analysis confirmed the two effects were jointly present in the frontal and occipital regions (p<0.001, corrected cluster threshold) and, to a lesser degree, in the precuneus (uncorrected cluster threshold). As seen in Figure 5, brain areas that showed greater Combined activity relative to averaged single feature activity overlapped with the more extensively distributed load-related network *(Medium > Low)*. The parameter estimate plots (Figure 4) illustrate that these two effects were expressed independently and additively in the common brain regions. For completeness, the possibility of an additive effect at encoding was investigated using the same statistical conjunction approach. No brain regions were observed to be jointly activated for both effects.

At retrieval, no regions reached statistical significance for *Combined > (Picture + Position).* No significant activity was observed at the whole brain level or with ROI analyses for *Combined < (Picture and Position)* at any task phase thus no brain regions were associated with reduced activity in the Combined task relative to single feature tasks.

A supplementary analysis comparing the Combined task with each single feature condition separately was performed to allow comparison to previous studies of feature binding independent of load [13], [18] (Table S2). Subtraction of the Position component alone or the Picture component alone from the Combined task typically revealed strong effects in regions characteristically engaged for the missing feature component, although there were some exceptions to this general trend at various phases of the WM trial. Conversely, subtraction of the Combined task from either single feature task, revealed limited activity in a few posterior regions that were preferentially engaged for visual processing of the relevant feature component.

#### Load effects on feature conjunction activity

**Figure 6:**
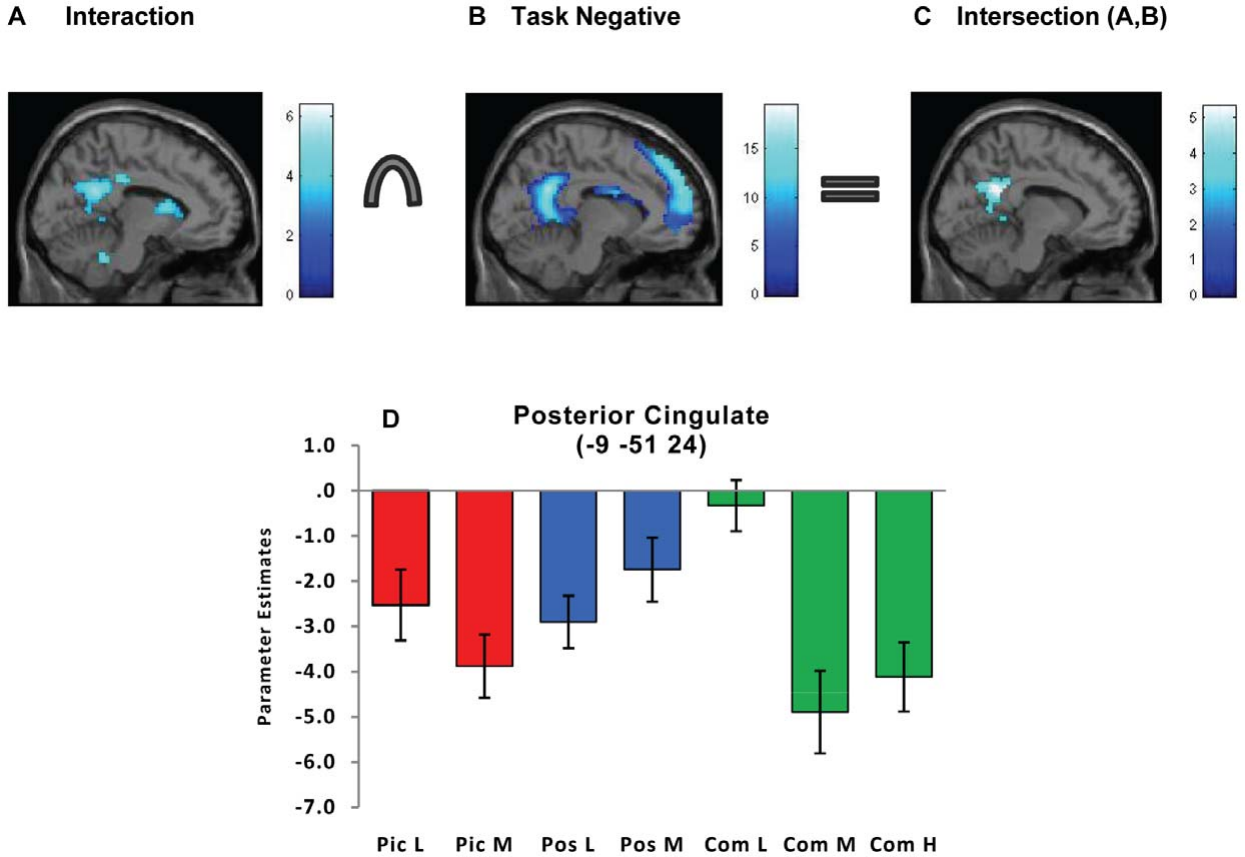
Interaction between task and load at retrieval. L =  Low load, M =  Medium load, H  =  High load, Pic  =  Picture task, Pos  =  Position task, Com  =  Combined task. **A.** Group mean activity for the task x load interaction at retrieval (Combined < averaged [Picture + Position] x Medium > Low) is superimposed on the SPM high resolution single subject T1-weighted image. Activity depicted is significant using a cluster-defining whole brain threshold of p<0.001 and FWE (p<0.05) cluster correction. **B.** A task-negative mask was created by applying a contrast isolating negative responses (below baseline) for all (task and load) conditions. Group mean activity is superimposed on the SPM high resolution single subject T1- weighted image. Activity depicted is significant using a cluster-defining whole brain threshold of p<0.001 and FWE (p<0.05) cluster correction. **C.** An inclusive mask was applied to form an intersection of regions common to the interaction effect and the task-negative mask. Group mean activity is superimposed on the SPM high resolution single subject T1- weighted image. Activity depicted is significant using a cluster-defining whole brain threshold of p<0.001 and FWE (p<0.05) cluster correction. **D.** Mean parameter estimates (β coefficients) for each task and load are shown for the peak voxel in the posterior cingulate cluster observed following inclusive masking of the task x load interaction and the set of task-negative regions (at corrected cluster threshold) shown in Figure 6C. As depicted in the plot, greater deactivation is observed between Low and Medium loads for the Combined task relative to the Picture and Position tasks. The beta estimate for the High load are also included in the figures to depict relative effects between load levels in the Combined task, however the main task effects were based on Low and Medium loads. Error bars represent ±1 standard error of the mean.

Task x load interaction t-contrasts were investigated in both directions to investigate directionality of the significant interaction revealed by the ANOVA. A strong task by load interaction effect *(Combined < [Picture + Position]) x (Medium > Low)* was observed in medial posterior regions of the limbic and parietal lobes during retrieval (see Table 2 and Figure 6A). The largest suprathreshold cluster was in the cingulate cortex, incorporating the posterior cingulate and medial precuneus. These regions have been reported to be components of the DMN [32].

Motivated by this observation, we undertook further analysis to establish whether the observed interaction effect was present in regions where activity is typically suppressed during cognitive task performance since task-related deactivation has been found to be a neural correlate of the DMN. First, a ‘task-negative mask’ was created by applying a contrast that defined a relative negative effect compared to the implicit baseline across all task and load conditions. The ‘task-negative mask’ activity (Figure 6B) resembles the well-established DMN [see examples in 35,36]). Second, the intersection of regions common to the interaction effect and this task-negative mask was estimated using an inclusive mask. Most of the cingulate region observed for the task x load interaction remained significant (peak voxel −9 −51 24, posterior cingulate BA 31; *T* = 5.33; cluster size  = 403) (Figure 6C). Inspection of the plot of the parameter estimates for posterior cingulate peak voxel (Figure 6D) suggests a relatively greater change in negative responses or deactivations for the Combined task as load was increased from Low to Medium while in comparison, task-related deactivation was less responsive to increased load for each single feature task. We addressed the possibility that the interaction effect may have been predominantly driven by weaker deactivation for the Combined task at Low load (see Figure 6D) by conducting a supplementary analysis of between-task effects at Low load. One small area in posterior cingulate (−12 −51 27) was observed at the whole-brain level (t = 4.33, cluster size  = 39, cluster-level corrected). Hence the apparent effect restricted to the low load is substantially smaller than the overall factorial effect across both levels.

To explore the relationship between load-dependent deactivation and task performance, Pearson correlations were performed between change in parameter estimates from the posterior cingulate over load levels and the corresponding change in accuracy (dPrime) over load levels. Consistent with our observations above, a significant correlation was observed between deactivation magnitude and accuracy (dPrime), as load increased, for the Combined task (r = 0.55, p = 0.02) but not for Picture (r = 0.21, p = 0.40) or Position (r = 0.20, p = 0.44) tasks.

In addition to the posterior cingulate, the interaction between load and feature conjunction was observed in a number of other regions considered part of the DMN [35], [37] namely lateral superior temporal cortex (63 0 −6) and anterior medial regions (uncorrected) – bilateral superior frontal (BA 9) and medial frontal (BA 10). The interaction effect was also present in the right caudate (21 15 15) which is functionally connected to parts of the DMN [38]–[40]. A similar pattern in the parameter estimates was observed in these regions, namely a strong load-related decrease in activity from Low to Medium in the Combined task relative to single feature tasks.

No other interaction effects were observed at the whole brain level or after ROI analyses. Thus, we did not obtain evidence of *positive* BOLD activity associated with the Combined task (compared to single feature tasks) when load was increased.

#### Effects restricted to correct trials only

The data were also characterized using a model restricted exclusively to correct trials since activity associated with correct and incorrect trials has been found to differ during different phases of a WM task [41]. Findings for correct trials were almost identical to the original analysis based on all trials (data not shown).

## Discussion

To the best of our knowledge, the present study is the first to directly investigate the neural correlates of binding in WM across the whole brain under conditions of increased load. Notably, the relationship between feature conjunction and load, as measured by changes in associated BOLD responses, varied according to the phase of the task. Firstly at encoding, specific effects were associated with task demands for feature conjunction relative to single features, independent of load. Secondly, during maintenance, the effects of feature conjunction and load were independent and were expressed additively in overlapping brain regions. Thirdly, at retrieval, an interaction effect suggested interdependence between feature conjunction and load for this phase of the task expressed by increased load-dependent deactivation in medial cortical structures. These differences at the neural level were present in the context of equivalent performance for feature conjunction and single feature conditions.

Our overarching objective was to investigate whether binding in WM is an efficient means of information compression as suggested by early fMRI research [3] or whether it is a resource-intensive process. Existing cognitive and neurophysiological models of binding make different assumptions about whether binding places additional demands on the cognitive or neural system. Based on these assumptions, we formulated predictions regarding the presence or absence of an interaction between conjunction and load on neural and behavioural responses. In terms of task performance, no interaction of conjunction and load was evident - participants were able to remember both features as well as single features, even under conditions of increased load. Similarly, no interaction was evident in the neural responses, for the encoding and maintenance phases of the task. The observed increase in activity in PFC and hippocampus during encoding, for the Combined task relative to single feature tasks, independent of load, is consistent with a number of other studies investigating the neural correlates of binding in WM [3], [12], [13], [15], [42]. While most studies have focused on the maintenance phase, our findings and others [42] suggest that these regions are also important for encoding multiple aspects of an object in a feature conjunction task. During the maintenance phase, effects related to increased load, and to feature conjunctions versus single features, were independently represented in overlapping left-sided regions of precuneus, superior occipital and superior frontal cortex. There was no apparent interaction, rather, activity that separately related to the two factors increased in an additive manner suggesting that these processes did not make joint demands on WM. In sum, the present findings suggest that cognitive processes engaged for encoding and maintaining bound representations do not place additional demands on the limited capacity WM system.

The absence of a conjunction by load interaction in neural responses at encoding and maintenance and in task performance is consistent with our predictions based on neurobiological models of binding. According to the temporal synchrony model, binding occurs automatically via synchronization of oscillating neural firing in specialised cortical feature maps (e.g. spatial and object) in visual cortex. Load would not be expected to modulate this process. According to the *modified* biased competition model, the PFC plays a role in biasing signals from the posterior visual processing streams in response to task demands. When maintaining conjunction items, competition between biasing signals from the two visual processing inputs leads to attenuation of responses in both types of selective feature cells within PFC. According to this model, the “load” of bound and single feature representations is the same. Therefore manipulation of load in our study would not be expected to differentially affect neural or behavioural responses for conjunctions and features. However the *increased* activity for conjunctions compared to single features observed in frontal cortex and hippocampus during encoding, and in frontal, parietal and occipital cortex during maintenance, is not consistent with predictions from either of the neurobiological models. On the other hand, in Baddeley's WM model, active higher level attention processes are assumed to play a role in the maintenance of bound representations in the episodic buffer [11]. One potential neural correlate of the episodic buffer may be the PFC as suggested by one of the earliest fMRI studies of binding in WM. In the study by Prabhakaran et al [3], increased PFC activity for integrated (letter and position) representations relative to separated representations was associated with better performance, leading to the proposition that binding provides an efficient WM representation. In our study and others [13], [16], [42], increased activity for conjunctions versus single or separated features, in PFC and other regions including parietal cortex and hippocampus, did not incur a behavioural advantage when conjunction probe trials required exact matching of combination of features. Indeed, when conjunction memory was more directly evaluated in a similar paradigm to that developed by Prabhakaran, Luck et al [42] observed increased activity for bound versus separate information in multiple brain areas including medial temporal lobes, parietal and frontal cortex in the context of worse performance. The authors suggest that these findings are compatible with the concept of the episodic buffer, since binding of verbal and spatial information made additional demands on attentional resources. The absence of an interaction of conjunction and load in neural responses during encoding and maintenance, and in performance in our study, however, suggests that cognitive processes engaged for object-location binding do not utilise additional WM resources during these phases of the task.

During the retrieval phase, however - when active processes involve the comparison of test stimuli to memory representations - an interaction of task and load was observed, consistent with Baddeley's cognitive model. Intriguingly, this interaction effect was expressed by greater deactivation in key regions of the DMN [32], [36], [43] and not in spatially localised areas of activation. The DMN is hypothesised to have a role in general monitoring of internal mental state and external environment [32], [44] and during performance of challenging cognitive tasks these processes are relatively deactivated [37], [45], [46]. In this study, greater deactivation in response to increased load (low to medium) was observed for all tasks and trial phases in regions corresponding to the DMN (Figure S2), consistent with previous findings that magnitude of deactivation is proportional to task demands such as WM load and task difficulty [47]–[52]. The task by load interaction present during retrieval in areas of the DMN, suggested that deactivation under conditions of increased load, differed according to whether the task made concurrent demands for feature binding. At low loads, relatively little deactivation was evident (Figure 6D) and so we propose that retrieval of feature conjunctions in this context presents minimal workload and may be relatively automatic. As load increased, however, deactivation became substantially stronger suggesting that retrieval of feature conjunctions had become more resource-demanding with load, resulting in relatively greater suppression of monitoring processes in DMN. In comparison, load did not modulate deactivation to the same degree in the context of single tasks. Intra-subject brain-behaviour associations support this interpretation. Subjects who more strongly deactivated posterior cingulate, a core region of the DMN as load increased during the conjunction task, also showed a greater decline in accuracy, a finding consistent with previous studies [51]. In contrast, change in deactivation between low and medium loads was not significantly correlated with accuracy for the single feature tasks in this region.

We considered the possibility that minimal deactivation in the Combined task at low load (see Figure 6D) was predominantly driving the interaction. However, analysis of task effects (Picture and Position < Combined) at low load only, revealed a substantially weaker effect restricted to a small area of posterior cingulate. This suggests that the interaction effect was not predominantly driven by the smaller deactivation at low load in the Combined task although this effect partially contributes to the overall interaction. Hence, we favour an interpretation in terms of relatively increased workload for retrieval of feature conjunctions when moving from low to higher load compared to single features, leading to greater reallocation of general cognitive resources from default mode to task-relevant processes, as suggested by the default mode hypothesis [53]. Although there is some expectation that reciprocal increased task-positive activity related to feature conjunction at retrieval would also be observed, there is no *a priori* reason to expect an interaction in one direction to be matched by a reciprocal effect, since interactions such as this do not correspond to “activations” but only to relative difference in effects across conditions. Furthermore, responses in task-positive networks are not necessarily proportional to responses in task-negative networks [e.g. 51] and any putative reciprocal interaction may have been expressed across a spatially distributed network that did not survive explicit cluster-wise correction.

Specific characteristics of the retrieval phase may explain why an interaction effect was seen only during this part of the task. During retrieval, probe stimuli need to be compared to stored representations. Behavioural studies suggest that bound representations held in WM may be more fragile than single feature representations [9] and may be more easily disrupted when concurrent demands on spatial attention are high [10], [54]. Hence, the increased demand on visuospatial attention going from a single probe at Low load to multiple probes at Medium load in this experiment may increase the overall cognitive challenge to a larger degree for the Combined task relative to single feature tasks, resulting in greater deactivation.

The high load was included in the Combined task since we were also interested in investigating supracapacity load-related responses. We found that load-related effects were attenuated beyond four targets as seen by a plateau or reduction in brain responses between Medium (N = 4) and High (N>4) loads in the context of a decline in performance. This was a general effect for all task phases and was observed in regions displaying positive and negative load-related responses (Figure S2). Previous studies have demonstrated WM capacity limits at high loads in task-positive brain regions [22], [29]–[31]. In this study we have shown the same capacity-constraints in terms of decreases in activity in DMN.

### Study limitations

A number of limitations of the present study require consideration. As with any functional imaging experiment, there is a trade-off between the number of arms of a factorial design that can be populated and the length of time that subjects can maintain concentration in a scanner without compromising their performance. Ideally we would have liked data from the full 3x3 design, as the requirements for binding would likely be different at supracapacity loads, but time constraints prohibited this. We would argue, though, that the load levels we did explored range across an important part of our day-to-day WM demands. Examination of cortical responses at the High level in the Combined task suggests only a weak although variable difference, with slightly less engagement of many regions at the High than at the Medium load. However, it is not possible to extrapolate these observations to effects of binding. A second important consideration concerns our employment of a WM trial with a fixed internal temporal structure. That is, although the inter-trial period was jittered in order to decorrelate subsequent trials, we did not jitter and hence decorrelate the components of a single trial. To do so would have required significantly longer maintenance periods (of up to 14 seconds) making successful completion of the task itself untenable for the longer maintenance trials. In addition, given that the nature and cortical location of WM processes differ according to the length of the maintenance period [55] this additional task component could not have been properly accounted for as a simple confound but would have required another explicit task factor, compounding our existing 2 (load) ×3 (task) factorial design. Because of such reasons this same limitation is found in many event-related fMRI studies of perceptual WM [e.g. 56–60]. However, it is critical to note that we did not perform contrasts between these different within-trial processes (i.e. between, say encoding and retrieval). All contrasts are between trial types, which differed according to the appropriately jittered experimental factors of load and/or feature conjunction. We do informally consider between-trial contrasts that differ according to the phase of the trial, noting that the slow BOLD response is likely to obscure possible differences between cortical responses in these different phases, rather than inflate or artificially create the subtle but nonetheless important differences that we have reported. Indeed, we formally investigated this statement by undertaking numerical integration of the predicted neurovascular response throughout our trial structure [61] in the presence of neuronal and measurement noise (Figure S1). Hence we observe that when true (i.e. simulated) differences are confined to one phase, the time series are likewise only different during this phase and return to within the noise-induced error bounds shortly thereafter. Conversely, when the true (simulated) effects are present throughout an entire trial the time series remain suitably separated. These simulations and time series visualizations provide face validity and theoretical support for our approach and are consistent with empirical studies that have shown that even four seconds spacing is sufficient to be able to uniquely resolve conjoint regressors such as those used in this experimental design [62]. Future studies, perhaps employing combined EEG-fMRI acquisitions to allow both spatial and temporal analyses, may be able to further disentangle distinct features of each WM component.

### Conclusion

In this study we have added an additional dimension to the existing literature on feature binding by implementing a factorial design to investigate the modulation of binding processes with WM load. Our findings provide new information on some of the mechanisms that mediate this relationship during WM performance. In particular, we report that this relationship qualitatively differs through different phases of WM, being predominantly additive during maintenance, pronounced and interactive during retrieval, and - at least in our data - not evident during encoding. This speaks to the complex relationship between binding demands, WM load and the differing computational demands of the different phases of a WM task. Of particular note, the interaction effect during retrieval reflected greater deactivation when moving from low to medium levels of load in the Combined compared to the single feature tasks. These findings suggest that in future studies, the relationship between feature binding and WM load needs to be interpreted in the context of the phase of the WM task.

## Supporting Information

Figure S1 **Comparison of exemplar experimental time series to numerically simulated BOLD responses obtained by integrating hemodynamic dynamics over the input structure of the working memory trial in the presence of system and measurement noise.**
(TIF)

Figure S2 **Load-related activity at each task phase.** Upper panel: Load-positive activity. Group mean activity for the contrast: Medium > Low at each phase of the task. Mean parameter estimates (β coefficients) are plotted for each task and load condition for the global maxima for each task phase; encoding, maintenance and retrieval. Lower panel: Load-negative activity. Group mean activity for the contrast: Medium < Low at each phase of the task. Regions were selected to demonstrate typical load-related negative responses although there was some variability in the distribution of the particular regions that were engaged within this network at different task phases. Medial frontal activity was more extensively distributed for the maintenance phase compared to encoding and retrieval (maintenance > encoding > retrieval) and posterior cingulate activity was more extensively distributed at retrieval relative to maintenance and encoding. Mean parameter estimates (β coefficients) are plotted for each task and load condition for voxels in suprathreshold clusters at each task phase. T-maps for each comparison are superimposed on the SPM high resolution single subject T1-weighted image. Activity depicted is significant using a cluster-defining whole brain threshold of p<0.001 and FWE (p<0.05) cluster correction. Error bars represent ±1 standard error of the mean.
(TIF)

Table S1 **Table listing regions of significant activity for** ***F*** **-contrasts of ANOVAs at encoding, maintenance and retrieval.**
(DOCX)

Table S2 **Table listing regions of significant activity for task components for each of the following contrasts: Picture>Position, Position>Picture, Combined > Picture, Combined > Position, Combined < Picture, Combined < Position at encoding, maintenance and retrieval.**
(DOC)
