# Supplementary figures and images for: Impact of Load-Related Neural Processes on Feature Binding in Visuospatial Working Memory
Source: PLoS One. 2011 Aug 24;6(8):e23960. doi: 10.1371/journal.pone.0023960 (PMC3161094; doi:10.1371/journal.pone.0023960)

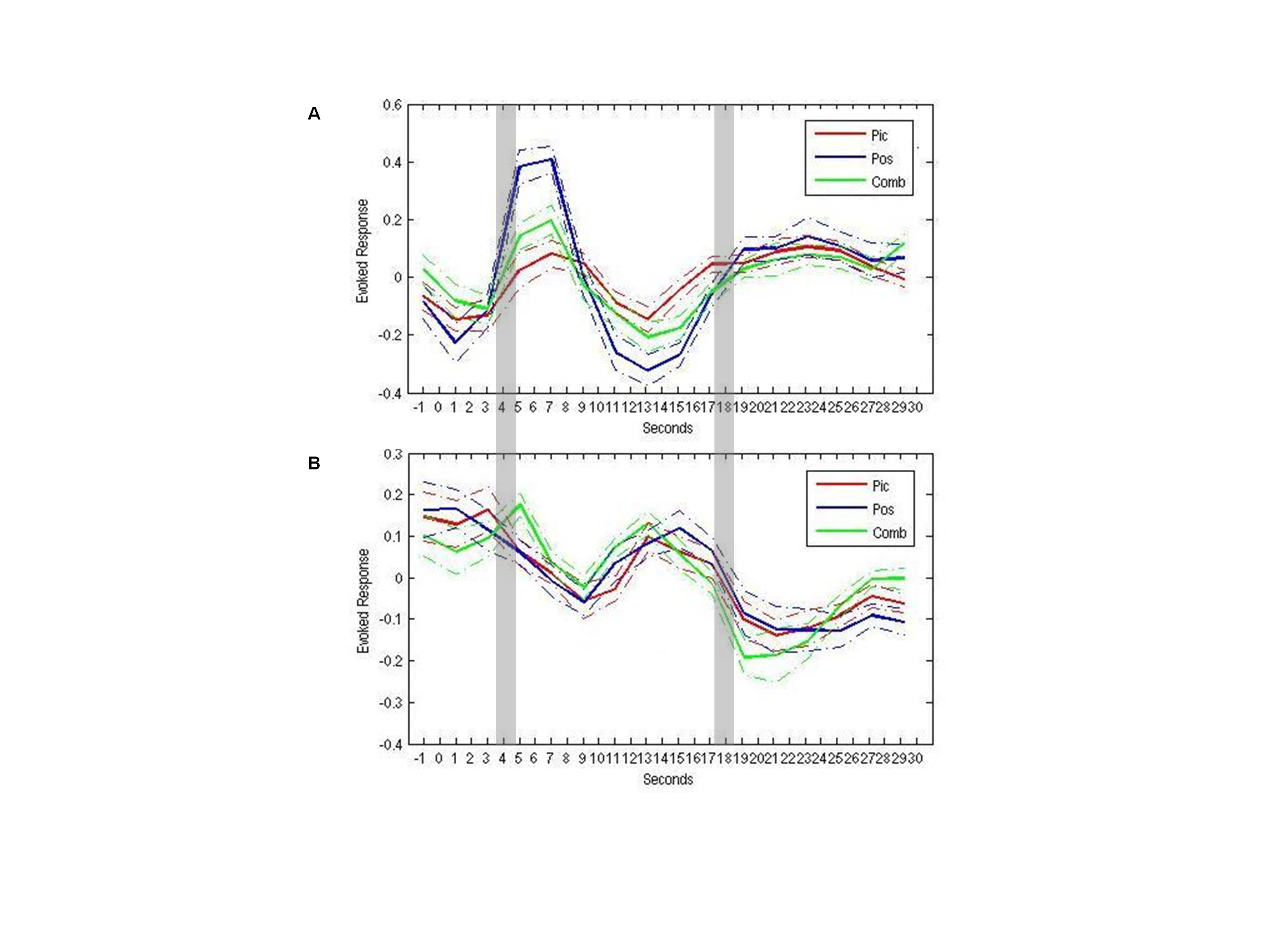

Supplement: Figure S1 — Comparison of exemplar experimental time series to numerically simulated BOLD responses obtained by integrating hemodynamic dynamics over the input structure of the working memory trial in the presence of system and measurement noise. (TIF) [file pone.0023960.s001.tif]

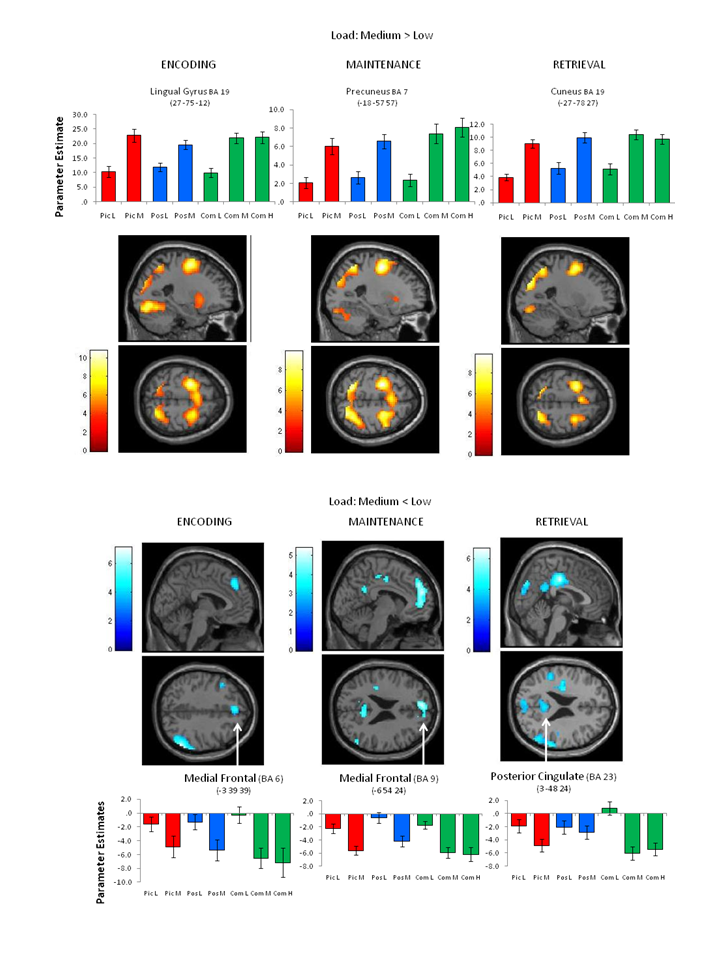

Supplement: Figure S2 — Load-related activity at each task phase. Upper panel: Load-positive activity. Group mean activity for the contrast: Medium > Low at each phase of the task. Mean parameter estimates (β coefficients) are plotted for each task and load condition for the global maxima for each task phase; encoding, maintenance and retrieval. Lower panel: Load-negative activity. Group mean activity for the contrast: Medium < Low at each phase of the task. Regions were selected to demonstrate typical load-related negative responses although there was some variability in the distribution of the particular regions that were engaged within this network at different task phases. Medial frontal activity was more extensively distributed for the maintenance phase compared to encoding and retrieval (maintenance > encoding > retrieval) and posterior cingulate activity was more extensively distributed at retrieval relative to maintenance and encoding. Mean parameter estimates (β coefficients) are plotted for each task and load condition for voxels in suprathreshold clusters at each task phase. T-maps for each comparison are superimposed on the SPM high resolution single subject T1-weighted image. Activity depicted is significant using a cluster-defining whole brain threshold of p<0.001 and FWE (p<0.05) cluster correction. Error bars represent ±1 standard error of the mean. (TIF) [file pone.0023960.s002.tif]
