# Supplementary material for: Impact of Load-Related Neural Processes on Feature Binding in Visuospatial Working Memory
Source: PLoS One. 2011 Aug 24;6(8):e23960. doi: 10.1371/journal.pone.0023960 (PMC3161094; doi:10.1371/journal.pone.0023960)
Supplement: Table S1 — Table listing regions of significant activity for F -contrasts of ANOVAs at encoding, maintenance and retrieval. (DOCX) [file pone.0023960.s003.docx]

**Table S1. Regions of Significant Activity for Factors of Task and Load in the visual WM Paradigm**

|  |  | **MNI coordinates** | | | | |  | |  |
| --- | --- | --- | --- | --- | --- | --- | --- | --- | --- |
| **Analysis** | **Brain region** | **Right/left** | **x** | ***y*** | ***z*** | ***F*-value** | **Brodmann Area** | |  |
| **Main Effect of Task** | | | | | | | | | |
| *Encoding* | Cuneus | R | 18 | -96 | 0 | 30.08 | 17 |  | |
|  | Lingual | R | 15 | 90 | -9 | 22.52 | 17 | |  |
|  | Fusiform | L | -45 | -57 | -15 | 25.71 | 37 | |  |
|  |  | L | -39 | -48 | -24 | 16.82 | 37 | |  |
|  | Cerebellum | R | 33 | -48 | -24 | 24.37 |  | |  |
|  |  | L | -30 | -45 | -24 | 18.05 |  | |  |
|  | Supramarginal Gyrus | R | 60 | -30 | 45 | 24.19 | 2 | |  |
|  | Inferior Parietal | R | 42 | -45 | 48 | 22.65 | 40 | |  |
|  | Precuneus | L | -6 | -72 | 48 | 22.09 | 7 | |  |
|  | Inferior Occipital | R | 36 | -90 | -6 | 18.67 | 18 | |  |
|  | Inferior Frontal | L | -45 | 30 | 12 | 18.38 | 46 | |  |
|  |  |  |  |  |  |  |  | |  |
| *Maintenance* | Cuneus | R | 9 | -69 | 6 | 33.51 | 30 | |  |
|  |  | L | -12 | -75 | 3 | 26.26 | 18 | |  |
|  | Lingual | L | -15 | -69 | -3 | 25.95 | 18 | |  |
|  | Middle Occipital | L | -30 | -72 | 27 | 18.78 | 19 | |  |
|  | Supplementary Motor Area | L | -9 | 12 | 51 | 17.35 | 32 | |  |
|  | Inferior Temporal | L | -45 | -42 | -15 | 16.78 | 37 | |  |
|  |  |  |  |  |  |  |  | |  |
| *Retrieval* | Supramarginal Gyrus | R | 57 | -27 | 45 | 30.23 | 2 | |  |
|  |  | R | 66 | -21 | 36 | 16.63 | 3 | |  |
|  | Superior Frontal | L | -21 | -3 | 60 | 25.18 | 6 | |  |
|  | Middle Frontal | L | -27 | 3 | 57 | 18.29 | 6 | |  |
|  |  | R | 27 | -9 | 51 | 18.86 | 6 | |  |
|  |  | R | 30 | -3 | 57 | 18.14 | 6 | |  |
|  | Precuneus | L | -15 | -60 | 57 | 22.39 | 7 | |  |
|  | Middle Occipital | R | 54 | -72 | 0 | 17.76 | 37 | |  |
|  | Middle Temporal | L | -48 | -57 | 3 | 16.63 | 37 | |  |
| **Main Effect of Load** | | | | | | | | | |
| *Encoding* | Lingual | R | 27 | -75 | -12 | 115.80 | 19 | |  |
|  |  | L | -15 | -87 | -9 | 56.49 | 18 | |  |
|  | Superior Frontal | L | -24 | -3 | 57 | 99.60 | 6 | |  |
|  | Fusiform | R | 27 | -63 | -12 | 98.95 | 19 | |  |
|  |  | L | -24 | -78 | -18 | 77.03 | 19 | |  |
|  | Inferior Parietal | R | 27 | -57 | 51 | 75.09 | 7 | |  |
|  | Cerebellum | L | -27 | -60 | -15 | 74.80 |  | |  |
|  |  | R | 6 | -30 | -9 | 53.65 |  | |  |
|  |  | R | 33 | -48 | -24 | 31.33 |  | |  |
|  |  |  | 0 | -57 | -42 | 38.14 |  | |  |
|  | Middle Frontal | R | 27 | 0 | 54 | 60.93 | 6 | |  |
|  |  | L | -42 | 15 | 42 | 28.62 | 9 | |  |
|  | Cuneus | R | 27 | -84 | 24 | 59.15 | 19 | |  |
|  | Caudate | L | -15 | 12 | 6 | 53.12 |  | |  |
|  | Supramarginal Gyrus | R | 60 | -54 | 36 | 51.18 | 40 | |  |
|  | Putamen | R | 18 | 15 | -9 | 50.11 |  | |  |
|  |  | R | 18 | 12 | 6 | 40.97 |  | |  |
|  | Supplementary Motor Area | L | -6 | 9 | 54 | 46.27 | 6 | |  |
|  |  | R | 3 | 6 | 63 | 45.73 | 6 | |  |
|  | Middle Occipital | L | -27 | -81 | 21 | 47.15 | 19 | |  |
|  |  | R | 42 | -81 | 12 | 33.53 | 19 | |  |
|  | Precuneus | L | -24 | -57 | 54 | 41.19 | 7 | |  |
|  |  | L | -15 | -60 | 57 | 35.57 | 7 | |  |
|  |  | R | 6 | -57 | 48 | 27.87 | 7 | |  |
|  | Inferior Frontal | L | -45 | 0 | 33 | 37.70 | 6 | |  |
|  | Insula | R | 45 | 6 | 0 | 33.65 | 13 | |  |
|  | Superior Occipital | R | 21 | -75 | 42 | 32.38 | 7 | |  |
|  | Superior Temporal | R | 48 | -3 | 3 | 29.21 | 22 | |  |
|  |  |  |  |  |  |  |  | |  |
| *Maintenance* | Superior Parietal | L | -18 | -57 | 57 | 96.84 | 7 | |  |
|  |  | R | 24 | -66 | 54 | 76.04 | 7 | |  |
|  |  | R | 30 | -57 | 60 | 73.10 | 7 | |  |
|  | Middle Frontal | L | -24 | -6 | 54 | 95.29 | 6 | |  |
|  |  | L | -45 | 3 | 39 | 54.60 | 9 | |  |
|  |  | R | 27 | 0 | 66 | 59.98 | 6 | |  |
|  |  | R | 45 | 0 | 54 | 47.99 | 6 | |  |
|  |  | R | 39 | 39 | 27 | 29.06 | 10 | |  |
|  | Superior Frontal | R | 24 | -3 | 51 | 75.41 | 6 | |  |
|  | Supplementary Motor area |  | 0 | 6 | 60 | 49.63 | 6 | |  |
|  |  | L | -3 | 3 | 69 | 37.56 | 6 | |  |
|  | Cuneus | L | -27 | -78 | 27 | 41.81 | 19 | |  |
|  | Inferior Frontal | L | -51 | 6 | 30 | 40.25 | 9 | |  |
|  | Cerebellum | R | 45 | -63 | -30 | 38.01 |  | |  |
|  |  |  | 0 | -78 | -27 | 29.52 |  | |  |
|  |  | L | -33 | -60 | -36 | 27.28 |  | |  |
|  | Caudate | L | -15 | 6 | 9 | 34.21 |  | |  |
|  |  | L | -15 | -3 | 18 | 27.75 |  | |  |
|  | Putamen | R | 24 | 18 | 3 | 33.55 |  | |  |
|  |  | L | 21 | -3 | 18 | 28.69 |  | |  |
|  | Inferior Parietal | R | 48 | -33 | 45 | 31.98 | 40 | |  |
|  | Middle Occipital | R | 30 | -81 | 27 | 30.09 | 19 | |  |
|  | Insula | L | -42 | -15 | 18 | 29.32 | 13 | |  |
|  | Fusiform | R | 24 | -87 | -21 | 29.06 | 19 | |  |
|  |  |  |  |  |  |  |  | |  |
| *Retrieval* | Cuneus | L | -27 | -78 | 27 | 86.38 | 19 | |  |
|  | Superior Parietal | R | 24 | -66 | 54 | 79.06 | 7 | |  |
|  | Middle Frontal | L | -24 | 0 | 57 | 63.81 | 6 | |  |
|  |  | L | -39 | 18 | 24 | 46.44 | 46 | |  |
|  |  | R | 30 | -3 | 57 | 42.54 | 6 | |  |
|  |  | R | 42 | 6 | 6 | 41.72 | 6 | |  |
|  | Precuneus | L | -18 | -60 | 54 | 54.71 | 7 | |  |
|  |  | R | 27 | -78 | 39 | 28.92 | 19 | |  |
|  | Medial frontal | L | -3 | 18 | 48 | 53.28 | 8 | |  |
|  | Inferior Frontal | L | -45 | 3 | 33 | 53.26 | 9 | |  |
|  |  | L | -30 | 24 | -3 | 31.73 | 47 | |  |
|  |  | R | 33 | 24 | -6 | 45.37 | 47 | |  |
|  | Middle Occipital | R | 30 | -81 | 24 | 50.97 | 19 | |  |
|  | Cingulate |  | 0 | -21 | 39 | 44.33 | 24 | |  |
|  | Supramarginal gyrus | R | 66 | -36 | 33 | 39.86 | 40 | |  |
|  | Lingual | L | -27 | -81 | -15 | 34.22 | 19 | |  |
|  | Middle Temporal | R | 60 | -45 | 0 | 32.21 | 21 | |  |
|  | Cerebellum | R | 6 | -30 | -9 | 30.19 |  | |  |
|  |  | L | -6 | -30 | -9 | 29.51 |  | |  |
|  | Inferior Parietal | R | 60 | -45 | 45 | 29.78 | 40 | |  |
| **Task x Load Interaction** | | | | | | | | | |
| *Retrieval* | Supramarginal gyrus | R | 60 | -27 | 42 | 23.62 | 3 | |  |
|  | Cingulate | L | -3 | -27 | 33 | 22.61 | 23 | |  |
|  | Caudate | R | 21 | 15 | 15 | 18.60 |  | |  |
|  | Putamen | R | 18 | 12 | 6 | 18.06 |  | |  |
|  | Posterior Cingulate | L | -9 | -51 | 24 | 17.97 | 31 | |  |
|  | Middle Temporal | R | 54 | -63 | -3 | 17.37 | 19 | |  |
|  |  |  |  |  |  |  |  | |  |

Significant voxels for F contrasts examining main effects for encoding, maintenance and retrieval, and interaction for retrieval (p<0.05 FWE-corrected whole brain threshold). Approximate Brodmann areas are listed.
