# Supplementary material for: Impact of Load-Related Neural Processes on Feature Binding in Visuospatial Working Memory
Source: PLoS One. 2011 Aug 24;6(8):e23960. doi: 10.1371/journal.pone.0023960 (PMC3161094; doi:10.1371/journal.pone.0023960)
Supplement: Table S2 — Table listing regions of significant activity for task components for each of the following contrasts: Picture>Position, Position>Picture, Combined > Picture, Combined > Position, Combined < Picture, Combined < Position at encoding, maintenance and retrieval. (DOC) [file pone.0023960.s004.doc]

|  |  | **MNI coordinates** | | | | |  |  |
| --- | --- | --- | --- | --- | --- | --- | --- | --- |
| **Analysis** | **Brain region** | **Right/left** | **x** | ***y*** | ***z*** | ***T*-value** | **Cluster size** | **Brodmann Area** |
| **Position > Picture** | | | | | | | | |
| *Encoding* | Cuneus | R | 18 | -96 | 0 | 7.46 | 134 | 17 |
|  | Lingual Gyrus | L | -15 | -93 | -12 | 5.56 | 50 | 17 |
|  | Inferior Parietal | R | 42 | -45 | 48 | 6.67 | 430 | 40 |
|  | Precuneus | L | -6 | -72 | 48 | 6.60 | 690 | 7 |
|  | Superior Frontal | L | -24 | -6 | 60 | 5.32 | 166 | 6 |
|  |  | R | 24 | 3 | 60 | 5.09 | 84 | 6 |
|  | Middle Frontal | R | 33 | 30 | 39 | 4.56 | 52 | 9 |
|  |  | L | -36 | 18 | 48 | 4.20 | 53 | 8 |
|  | Anterior Cingulate |  | -6 | 48 | -3 | 4.48 | 65 | 32 |
|  |  |  |  |  |  |  |  |  |
| *Maintenance* | ns |  |  |  |  |  |  |  |
|  |  |  |  |  |  |  |  |  |
| *Retrieval* | Middle Occipital | R | 54 | -72 | 0 | 5.95 | 146 | 37 |
|  | Supramarginal gyrus | R | 57 | -27 | 45 | 7.76 | 487 | 2 |
|  | Precuneus | L | -15 | -60 | 57 | 6.41 | 110 | 7 |
|  |  | R | 30 | -78 | 42 | 4.36 | 56 | 19 |
|  | Inferior Parietal | L | -54 | -30 | 30 | 5.26 | 133 | 40 |
|  | Middle Temporal | L | -42 | -69 | 9 | 4.90 | 76 | 39 |
|  | Superior Frontal | L | -21 | -3 | 60 | 7.06 | 263 | 6 |
|  | Middle Frontal | R | 27 | -9 | 51 | 6.11 | 267 | 6 |
|  |  | L | -36 | 42 | 24 | 4.50 | 53 | 10 |
|  | Inferior Frontal | R | 54 | 9 | 18 | 4.66 | 158 | 44 |
|  | Putamen | L | -24 | -6 | 6 | 4.50 | 72 |  |
| **Picture > Position** | | | | | | | | |
| *Encoding* | Fusiform | L | -45 | -57 | -15 | 5.75 | 166 | 37 |
|  | Cerebellum | R | 36 | -45 | -27 | 5.30 | 263 |  |
|  |  | L | -6 | -36 | -3 | 4.32 | 58 |  |
|  | Inferior Frontal | L | -45 | 30 | 12 | 5.63 | 159 | 46 |
|  | Superior Frontal | L | -6 | 15 | 60 | 4.55 | 56 | 8 |
|  | Cingulate | L | -3 | 3 | 24 | 4.06 | 50 | 24 |
|  |  |  |  |  |  |  |  |  |
| *Maintenance* | Cuneus | R | 9 | -69 | 6 | 7.66 | 1912 | 30 |
|  | Parahippocampal | L | -42 | -30 | -15 | 4.84 | 91 | 36 |
|  | Supplementary Motor area | R | 3 | 21 | 51 | 5.01 | 201 | 8 |
|  | Inferior Frontal | L | -45 | 27 | 15 | 4.95 | 143 | 46 |
|  | Thalamus |  | 0 | -12 | 12 | 3.89 | 50 |  |
|  |  |  |  |  |  |  |  |  |
| *Retrieval* | Middle Occipital | L | -12 | -90 | 12 | 4.10 | 67 | 18 |

**Table S2. Regions of Significant Activity related to Task Components**

|  |  | **MNI coordinates** |  |  |
| --- | --- | --- | --- | --- |

Table S2 continued

| **Analysis** | **Brain region** | | **Right/left** | **x** | ***y*** | ***z*** | ***T*-value** | **Cluster size** | **Brodmann Area** |
| --- | --- | --- | --- | --- | --- | --- | --- | --- | --- |
| **Combined > Picture** | | | | | | | | | |
| *Encoding* | ns | |  | | | | | | |
|  |  | |  |  |  |  |  |  |  |
| *Maintenance* | Superior Frontal | | L | -15 | -3 | 69 | 4.85 | 89 | 6 |
|  | Middle Frontal | | R | 30 | 0 | 57 | 4.80 | 92 | 6 |
|  |  | |  |  |  |  |  |  |  |
| *Retrieval* | Precuneus | | L | -9 | -60 | 54 | 4.98 | 121 | 7 |
|  | Supramarginal gyrus | | R | 54 | -30 | 42 | 4.16 | 59 | 2 |
|  | Inferior Temporal | | R | 54 | -57 | -6 | 4.52 | 65 | 37 |
|  | Middle Frontal | | R | 30 | -3 | 57 | 4.58 | 88 | 6 |
|  |  | | L | -27 | 0 | 60 | 4.51 | 91 | 6 |
|  |  | | L | -33 | 21 | 42 | 4.14 | 49 | 9 |
| **Combined > Position** | | | | | | | | | |
| *Encoding* | Fusiform | | L | -42 | -57 | -15 | 6.81 | 262 | 37 |
|  | Cerebellum | | R | 33 | -48 | -24 | 6.62 | 371 |  |
|  | Inferior Frontal | | L | -30 | 33 | -9 | 5.16 | 169 | 47 |
|  |  | |  |  |  |  |  |  |  |
| *Maintenance* | Superior Occipital | | L | -30 | -72 | 27 | 6.13 | 167 | 19 |
|  | Fusiform | | L | -45 | -42 | -15 | 5.43 | 151 | 37 |
|  | Cerebellum | | R | 39 | -63 | -33 | 4.40 | 139 |  |
|  | Supplementary Motor area | | L | -9 | 12 | 51 | 5.72 | 187 | 32 |
|  | Middle Frontal | | L | -27 | 42 | 6 | 4.79 | 155 | 10 |
|  |  | |  |  |  |  |  |  |  |
| *Retrieval* | ns |  | | | | | | | |
| **Combined < Picture** | | | | | | | | | |
| *Encoding* | ns | |  |  |  |  |  |  |  |
|  |  | |  |  |  |  |  |  |  |
| *Maintenance* | Cuneus | | R | 6 | -69 | 6 | 6.27 | 979 | 30 |
|  |  | |  |  |  |  |  |  |  |
| *Retrieval* | Lingual Gyrus | | R | 12 | -93 | -12 | 4.57 | 101 | 17 |
| **Combined <Position** | | | | | | | | | |
| *Encoding* | Supramarginal Gyrus | | R | 63 | -30 | 42 | 5.25 | 224 | 40 |
|  |  | |  |  |  |  |  |  |  |
| *Maintenance* | ns | |  |  |  |  |  |  |  |
|  |  | |  |  |  |  |  |  |  |
| *Retrieval* | ns | |  |  |  |  |  |  |  |

List of significant clusters for task comparisons averaged over Low and Medium loads for encoding, maintenance and retrieval phases. Standardized Montreal Neurological Institute (MNI) co-ordinates represent peak maxima of significant clusters (family-wise error (FWE)-corrected threshold). Approximate Brodmann areas are listed.
